# Safety analysis of German real-life cohort WIP shows rates of neuropsychiatric events leading to discontinuation of raltegravir therapy below 2%

**DOI:** 10.1177/0956462418812642

**Published:** 2019-05-21

**Authors:** U Naumann, A Moll, D Schleehauf, KT Lutz, W Schmidt, H Jaeger, B Funke, V Witte

**Affiliations:** 1 UBN/PRAXIS, Berlin, Germany; 2 Infektiologikum Frankfurt, Frankfurt, Germany; 3 MVZ Ärzteforum Seestrasse, Berlin, Germany; 4 MVZ Karlsplatz, HIV Research and Clinical Care Centre, Munich, Germany; 5 MSD Germany, Medical Affairs, Haar, Germany

Integrase inhibitors (INIs) are frequently used in routine clinical practice in Germany. Recently, discontinuation rates of 5% and beyond due to neuropsychiatric adverse events (AEs) and sleep disturbance have been reported from clinical cohorts for the INI dolutegravir.^1–3^

This post-hoc analysis describes patterns of discontinuation of the INI raltegravir (RAL) in the German real-life cohort WIP (efficacy of ISENTRESS® [Merck Sharp & Dohme Limited, GmbH, Haar, Germany] under routine clinical conditions), in particular those discontinuations due to AEs with a focus on neuropsychiatric and gastrointestinal events.

The WIP study was a prospective, observational, multicentre cohort study in routine clinical care with data collection between 2010 and 2014 in Germany. Safety and efficacy outcomes of RAL-based antiretroviral therapy (ART) in a population enriched for aging patients (total *N* = 451, ≥50 years: *n* = 274; 61%) were documented. Detailed methods and efficacy results have been published previously in this journal.^4^

The median time since HIV diagnosis was 10.5 (±8.2) years. The median observed duration of ART with RAL was 344 days (range 25–511 days); 382/451 patients (84.7%) were male, 96 (21.3%) were previously untreated.

Sixty-seven patients (*n* = 67/451; 14.9%) discontinued RAL during the observation period. With the possibility to report multiple reasons, the reasons for discontinuation were lack of efficacy (*n* = 26; 5.8%), AEs (*n* = 22; 4.9%), ART switch for unknown reasons (*n* = 19; 4.2%), poor compliance (*n* = 5; 1.1%) and other reasons (*n* = 34; 7.5%). There was a trend towards a higher risk of discontinuation due to any reason in patients ≥50 years (*n* = 47/274; 17.2%) vs. <50 years (*n* = 20/177; 11.3%). The difference did however not reach statistical significance. Regarding gender, no difference in discontinuation due to any reason was observed (male: *n* = 58/382; 15.2% vs. female: *n* = 9/69; 13.1%).

Of the 22 discontinuations due to AEs, only 2 (0.4%) occurred within the first 100 days of RAL therapy (abdominal pain, hepatic enzyme increase). In four patients, AEs were classified as unrelated to RAL: hepatic failure (*n* = 1) and cancer (*n* = 3). All four patients died within two days after discontinuation. The remaining 18 (4.0%) patients discontinued RAL due to AEs possibly related to RAL. In seven^a^ (1.6%) patients the documented reasons for discontinuation were neuropsychiatric disorders and in four^b^ (0.9%) patients gastrointestinal disturbances.

The proportion of discontinuations due to AEs did not differ by age group (≥50 years: *n* = 16/274; 5.8% vs. <50 years: *n* = 6/177; 3.4%) or gender (male: *n* = 18/382; 4.7% vs. female *n* = 4/69; 5.8%).

The findings from this analysis of observational data could be subject to channelling bias regarding the selection of a RAL-based regimen for patients with more comorbidities and concomitant medications or to reporting bias regarding AEs and their relationship to medication. During part of the observation period RAL was the only INI available in Germany, so that patients might have been more motivated to accept tolerability issues due to the lack of alternatives in the same drug-class. Further, comparisons across different cohort analyses should be interpreted with caution.

In this large German real-life cohort, discontinuations due to neuropsychiatric AEs potentially related to RAL-based therapy were observed infrequently. This is consistent with recently published data^5^ and considerably lower than observed with dolutegravir under real-world conditions in Germany.^1^

## Notes

a Neuropsychiatric events in seven subjects: headache (1), dizziness (4), mood swings (1), depressed mood (1), personality disorder (1), depersonalization (1), disturbance of attention (1).

b Gastrointestinal events in four subjects: abdominal pain (1), gastric ulcer haemorrhage (1), vomiting (1), anorectal discomfort (1), nausea (1), dyspepsia (1).
